# Reducing the burden of low back pain: results from a new microsimulation model

**DOI:** 10.1186/s12891-022-05747-2

**Published:** 2022-08-23

**Authors:** Jacek A. Kopec, Eric C. Sayre, Jolanda Cibere, Linda C. Li, Hubert Wong, Anya Okhmatovskaia, John M. Esdaile

**Affiliations:** 1 School of Population and Public Health, University of British Columbia, 2206 E Mall, Vancouver, BC V6T 1Z3 Canada; 2 Arthritis Research Canada, Vancouver, BC Canada; 3 Department of Medicine, University of British Columbia, Vancouver, BC Canada; 4 Department of Physical Therapy, University of British Columbia, Vancouver, BC Canada; 5 McGill Clinical and Health Informatics, McGill University, Montreal, QC Canada

**Keywords:** Low back pain, Years lived with disability, Body mass index, Occupation, Exercise, Microsimulation modeling

## Abstract

**Background:**

Low back pain (LBP) causes the highest morbidity burden globally. The purpose of the present study was to project and compare the impact of three strategies for reducing the population health burden of LBP: weight loss, ergonomic interventions, and an exercise program.

**Methods:**

We have developed a microsimulation model of LBP in Canada using a new modeling platform called SimYouLate. The initial population was derived from Cycle 1 (2001) of the Canadian Community Health Survey (CCHS). We modeled an open population 20 years of age and older. Key variables included age, sex, education, body mass index (BMI), type of work, having back problems, pain level in persons with back problems, and exercise participation. The effects of interventions on the risk of LBP were obtained from the CCHS for the effect of BMI, the Global Burden of Disease Study for occupational risks, and a published meta-analysis for the effect of exercise. All interventions lasted from 2021 to 2040. The population health impact of the interventions was calculated as a difference in years lived with disability (YLDs) between the base-case scenario and each intervention scenario, and expressed as YLDs averted per intervention unit or a proportion (%) of total LBP-related YLDs.

**Results:**

In the base-case scenario, LBP in 2020 was responsible for 424,900 YLDs in Canada and the amount increased to 460,312 YLDs in 2040. The effects of the interventions were as follows: 27,993 (95% CI 23,373, 32,614) YLDs averted over 20 years per 0.1 unit change in log-transformed BMI (9.5% change in BMI) among individuals who were overweight and those with obesity, 19,416 (16,275, 22,557) YLDs per 1% reduction in the proportion of workers exposed to occupational risks, and 26,058 (22,455, 29,661) YLDs averted per 1% increase in the proportion of eligible patients with back problems participating in an exercise program.

**Conclusions:**

The study provides new data on the relationship between three types of interventions and the resultant reductions in LBP burden in Canada. According to our model, each of the interventions studied could potentially result in a substantial reduction in LBP-related disability.

**Supplementary Information:**

The online version contains supplementary material available at 10.1186/s12891-022-05747-2.

## Introduction

Low back pain (LBP) has been the leading cause of disability worldwide for the past 30 years [1, 2]. Globally in 2019, LBP was responsible for 64 million years lived with disability (YLDs) [3]. In the US, LBP is ranked 3^rd^ in terms of disability-adjusted life years, behind ischemic heart disease and drug disorders [3]. LBP is one of the top reasons for seeing a doctor and a major cause of work absence and lost productivity [4]. Healthcare spending for low back and neck pain in the US has been recently estimated at $135 billion per year [5]. In Canada, the direct costs of LBP in Ontario in 2014/15 were $330 million [6].

Burden reduction strategies for LBP have evolved over the past 3 decades with a better understanding of the natural course of this condition and the effectiveness of various treatment and preventive approaches [1, 7]. It is now recognized that many people develop back problems relatively early in life, may experience many episodes of back pain over lifetime and be free from pain for extended periods of time [1, 8]. Point prevalence of LBP increases with age and is higher in women and among smokers [3]. Poor general and mental health and lower socioeconomic status are associated with LBP, although globally LBP is more common in high-income countries [1–3]. Established modifiable risk factors for LBP include high body mass index (BMI) and type of work [3]. Specific work-related factors associated with LBP are physically demanding work, frequent bending, heavy lifting, and whole-body vibration [9]. Many other risk factors have been reported, including stress, job dissatisfaction, low levels of social support, and sedentary lifestyle [9, 10]. In terms of secondary prevention, exercise is effective in chronic LBP [11, 12]. Other non-pharmacological therapies have been recommended but strong evidence of their effectiveness is lacking [7].

The purpose of the current study was to model the population health effects of different strategies for reducing the burden of LBP. We compared the effects of primary prevention through weight reduction and ergonomic interventions in the workplace, and secondary prevention through exercise for patients with back problems. These strategies were selected because they are feasible and relatively well studied. The effect of exercise is supported by data from randomized trials, whereas evidence for weight reduction and ergonomic interventions comes primarily from epidemiological studies [1–3, 7–12].

## Methods

### Population

We have developed a microsimulation model of low back pain in Canada using the SimYouLate (SYL) platform described in detail in Supplementary Materials. SYL is very flexible, continuous-time microsimulation software developed by one of the authors (ECS), designed specifically for modellers with no programming background and particularly suitable for population health modeling [13]. We modeled an open population 20 years of age and older from 2001 until 2040. Individuals were allowed to enter the population either by immigration or by becoming 20 years of age and exit by emigration or death. The initial population was derived from the 2001 cycle of the Canadian Community Health Survey (CCHS, *n* = 102,761 after age restriction) and was representative of the household population of Canada [14]. Age/sex-specific mortality rates were based on Statistics Canada’s demographic projections [15]. Rates of entry and exit over time by age and sex were obtained by calibration, using data from the 2001 and 2005 cycles of the CCHS, and validated in data from the 2016 cycle.

### Variables

The key variables in the model were age, sex, education, BMI (from self-reported height and weight), occupation, having back problems, and pain level (0–4) in persons with back problems. Occupational group was based on the National Occupational Classification Statistics (NOC-S) 2006 at the 1-digit level [16]. Survey respondents were asked if they had “back problems” that lasted or were expected to last 6 months or longer [14]. Future prevalence of chronic back problems was estimated from a logistic regression model with age, sex, BMI, and education (as a proxy for socio-economic status). These variables were chosen because they are established predictors of back problems in the general population [1–3, 9, 10]. We estimated age- and sex-specific incidence rates of new back problems by calibration, using our prevalence model and assuming life-long duration. We then modeled trajectories of pain over time in persons with back problems using the pain question in the CCHS, derived from the Health Utilities Index Mark 3 [17]. The levels of pain were: 0—no pain or discomfort; 1—mild to moderate pain that prevented no activities; 2—moderate pain that prevented a few activities; 3—moderate to severe pain that prevented some activities; and 4—severe pain that prevented most activities. For the base-case model, the probability of having pain at each level at time t for each simulated individual was calculated from an autoregressive ordinal logistic regression model with proportional odds, with age, sex, education, BMI, occupation, and pain at time t-1 (previous year) as predictors. Thus each person with back problems had a specific LBP trajectory. The model assumed that all patients newly diagnosed with back problems had pain initially; however, as their subsequent level of pain was simulated, they could be free from pain for periods of time and experience new episodes of pain. The autoregressive component of the model was obtained from an analysis of the longitudinal National Population Health Survey in Canada, which followed a sample of over 17,000 persons from 1994 until 2012 [18].

### Interventions

We considered three interventions, a weight reduction intervention, an ergonomic intervention, and an exercise intervention (Table A2-1, Supplementary Materials). We evaluated 10 to 18 scenarios per intervention plus the base-case scenario, for a total of 39 scenarios. For each intervention type, the scenarios differed in the level (intensity or coverage) of the intervention and the target population group. For the weight reduction intervention we assumed an average reduction in BMI from 0.1 to 5 BMI units per year for all persons with BMI ≥ 25. For the ergonomic interventions, we considered interventions whereby the risk of LBP associated with occupational exposures would be eliminated in a proportion (ranging from 0 to 100%) of all active workers or, alternatively, the risk would be eliminated in all workers in a specified occupational group. For the exercise intervention, we assumed that a proportion of persons in the target group, ranging from 0 to 100%, would receive exercise therapy that may include muscle stretching and strengthening as well as endurance, posture, balance and/or aerobic exercises, with or without education. The target group for this intervention was persons less than 80 years of age with pain level < 4 (severe pain that prevents most activities). All interventions were assumed to be fully implemented in 2021 and lasted until 2040. In the weight reduction intervention, we assumed a gradual reduction in weight by a specified amount per year, whereas in the ergonomic and exercise interventions we assumed that a specified, constant proportion of the target group would obtain the intervention each year.

**Table 1:** Comparison of the distribution of key variables in the CCHS in 2016 and in SYL in 2016, 2020 and 2040: base-case scenario

Variable	SYL 2016	SYL 2020	SYL 2040	CCHS 2016
Age 20–29 (%)	11.5	11.1	10.33	16.6
Age 30–39 (%)	21.1	17.9	15.73	18.0
Age 40–49 (%)	16.6	18.76	15.56	17.6
Age 50–59 (%)	21.4	19.47	18.03	19.0
Age 60–69 (%)	15.6	17.91	17.56	15.8
Age 70–79 (%)	9.3	10.23	13.93	8.8
Age 80 + (%)	4.5	4.63	8.85	4.2
Male sex (%)	48.5	48.4	47.5	49.1
BMI (mean)	25.9	25.9	26.0	26.4
Back problems (%)	20.4	20.7	20.9	21.9
Pain level 0 (%)	56.7	57.7	58.0	55.5
Pain level 1 (%)	9.8	10.3	9.8	9.7
Pain level 2 (%)	12.2	11.6	12.1	13.5
Pain level 3 (%)	12.5	11.4	11.6	13.9
Pain level 4 (%)	8.9	9.0	8.6	7.4

Levels of pain: 0—no pain or discomfort; 1—mild to moderate pain that prevented no activities; 2—moderate pain that prevented a few activities; 3—moderate to severe pain that prevented some activities; and 4—severe pain that prevented most activities. *CCHS* Canadian Community Health Survey, *SYL* SimYouLate, *BMI* body mass index

**Table 2:** YLDs averted per unit of intervention: estimates from linear regression models

Intervention (units)	YLDs averted per unit of intervention, 95% CI		
	**Estimate**	**LCL**	**UCL**
Reduction in log-transformed BMI (0.1)	27,993	23,373	32,614
Reduction in occupational risk (1%)	19,416	16,275	22,557
Exercise participation (1%)	26,058	22,455	29,661

For the BMI intervention the unit is 0.1 log BMI. For the occupational and exercise interventions, the unit is 1.0%. *YLD* Years lived with disability, *BMI* Body mass index, *CI* Confidence interval, *LCL* Lower 95% confidence limit, *UCL* Upper 95% confidence limit

### Parameters

The adjusted odds ratio for the effect of BMI, treated as a continuous variable, on pain level in the CCHS was OR = 1.14 (95% CI 1.11 to 1.17) per 5 BMI units (Table A2-2, Supplementary Materials). This effect was considered the same for all age/sex groups. The effect of occupation (occupational category) was obtained directly from the Global Burden of Disease Study (GBD), using the mean value of relative risk (RR) across all age groups [19]. The RRs ranged from 1.0 for Group 3 (clerical and related workers) to 3.78 (2.61 to 5.31) for Group 6 (agriculture, animal husbandry and forestry workers, fishermen and hunters). We have mapped the GBD occupational groups to the classification of occupations used in the CCHS (Table A2-3, Supplementary Materials). The effect of exercise was derived from a published meta-analysis of exercise trials in which the relative risk associated with exercise (with or without education) vs. usual care was estimated as RR = 0.71 (0.60 to 0.83) [11]. In addition, as a form of model validation, we specified the effect of exercise as an average reduction in pain level from a comprehensive review of non-invasive treatments for LBP, estimated as 10 units (1.31 to 19.09) on a 0–100 visual analog scale (VAS) [11] (Table A2-2, Supplementary Materials).

**Table 3:** YLDs averted between 2021 and 2040 for three types of interventions

Intervention	Total YLDs averted, 95% CI			YLDs averted as % of all LBP-related YLDs, 95% CI		
	**Estimate**	**LCL**	**UCL**	**Estimate**	**LCL**	**UCL**
Mean BMI reduction per year						
0.1	124,220.3	-124,051.7	372,492.3	1.4	-1.4	4.1
0.3	431,760.0	201,178.3	662,341.7	4.8	2.2	7.3
0.5	574,757.8	348,960.5	800,555.0	6.3	3.9	8.8
1.0	768,793.7	545,627.2	991,960.2	8.5	6.0	11.0
2.0	962,829.6	737,721.3	1,187,937.9	10.6	8.1	13.1
3.0	1,076,333.3	848,004.7	1,304,661.9	11.9	9.4	14.4
4.0	1,156,865.5	925,358.0	1,388,373.0	12.8	10.2	15.3
5.0	1,219,331.1	984,868.0	1,453,794.2	13.5	10.9	16.0
Reduction in occupational risk for all groups combined						
20%	472,775.4	173,700.9	771,850.0	5.2	1.9	8.5
40%	861,090.3	575,514.9	1,146,665.7	9.5	6.4	12.7
60%	1,249,405.2	963,829.8	1,534,980.6	13.8	10.6	16.9
80%	1,637,720.1	1,338,645.6	1,936,794.7	18.1	14.8	21.4
100%	2,026,035.0	1,701,643.1	2,350,426.9	22.4	18.8	25.9
Elimination of occupational risk by occupational group						
Group 1	472,776.2	173,845.7	771,706.6	5.2	1.9	8.5
Group 2	210,055.0	-105,132.3	525,242.2	2.3	-1.2	5.8
Group 4&5	736,558.2	447,954.8	1,025,161.6	8.1	4.9	11.3
Group 6	510,344.5	213,251.7	807,437.3	5.6	2.4	8.9
Group 7	821,772.5	535,221.4	1,108,323.6	9.1	5.9	12.2
Exercise participation (effect measured by RR)						
20%	597,534.2	254,446.2	940,622.1	6.6	2.8	10.4
40%	1,118,694.9	791,092.6	1,446,297.1	12.4	8.7	16.0
60%	1,639,855.6	1,312,253.3	1,967,457.8	18.1	14.5	21.7
80%	2,161,016.3	1,817,928.3	2,504,104.3	23.9	20.1	27.6
100%	2,682,177.0	2,310,045.8	3,054,308.1	29.6	25.5	33.7
Exercise participation (effect measured by VAS)						
20%	590,639.2	423,560.4	757,718.1	6.5	4.7	8.4
40%	1,143,701.5	984,164.0	1,303,239.1	12.6	10.9	14.4
60%	1,696,763.8	1,537,226.3	1,856,301.4	18.7	17.0	20.5
80%	2,249,826.1	2,082,747.2	2,416,905.0	24.8	23.0	26.7
100%	2,802,888.4	2,621,665.9	2,984,110.9	30.9	28.9	32.9

The occupational groups are: 1—Professional, technical and related workers, 2—Administrative and managerial workers, 3—Clerical and related workers, 4—Sales workers, 5—Service workers, 6—Agriculture, animal husbandry, and forestry workers, fishermen and hunters, and 7—Production and related workers, transport, equipment operators and laborers. Group 3 is not shown since RR = 1.0 for this group. *YLDs* Years lived with disability, *LBP* Low back pain, *BMI* Body mass index, *RR* Relative risk, *VAS* Visual analog scale, *CI* Confidence interval, *LCL* Lower 95% confidence limit, *UCL* Upper 95% confidence limit. Please see Table A2-1 (Supplementary Materials) for a detailed description of each intervention

### Outcomes

The effects of interventions were assessed in terms of years lived with disability (YLDs) due to LBP for the entire adult population. YLDs were calculated as prevalence of each level of pain multiplied by the appropriate disability weight [20]. We used the disability weights for LBP developed by the GBD. The weights were developed through a series of large, international population-based studies [21]. We mapped the GBD LBP severity levels to pain levels in the CCHS (Table A2-4, Supplementary Materials). We estimated YLDs for each year between 2021 and 2040 for all scenarios. We obtained the effect of each intervention, defined as YLDs averted and calculated as a difference between base-case YLDs and each intervention scenario’s YLDs, for each year. Finally, we calculated the cumulative YLDs averted as a sum of YLDs averted over the years 2021 to 2040. Because of stochastic variation in the model, we smoothed the relationship between the level of each intervention and YLDs using appropriate statistical regression models.

The results were expressed as total YLDs averted (counts), YLDs averted as a fraction (%) of total LBP-related YLDs, and changes in YLD rates per 1000 person-years. We compared the interventions through an equivalence plot, which shows the levels of different interventions resulting in the same numbers of YLDs averted. Equivalence values were obtained by calculating intervention levels (intensity or coverage) required to avert specified numbers of YLDs from the regression equations. For all estimates we provide 95% confidence intervals from the regression models.

## Results

### Sample description

In the simulation model, the initial sample was 102,761 individuals. We applied survey weights to obtain the results for the population of Canada aged 20 years and older (~ 27,000,000 in 2020) from 2001 to 2040. In 2020, 32.8% of the simulated subjects were aged ≥ 60 years, 51.6% were females, and 52.2% had college education (Table 1). The mean BMI was 25.9, and 20.7% had back problems. Among those with back problems, the proportions in pain levels 0, 1, 2, 3, and 4, were 57.7%, 10.3%, 11.6%, 11.4% and 9.0%, respectively. In 2040, the proportion of persons aged ≥ 60 years increased to 40.3%, 52.5% were females, average BMI was 26.0, 20.9% had back problems, and the distribution of pain levels remained largely unchanged.

### Model validation

The distribution of demographic variables, BMI, and LBP in the CCHS and SYL samples in 2016 are compared in Table 1. For example, the proportion of persons aged ≥ 60 years was 28.8% in the CCHS and 29.4% in SYL. The proportion with chronic back problems was 21.9% in the CCHS and 20.4% in SYL, and among those with chronic back problems, 44.5% had pain at the time of the survey in the CCHS compared with 43.3% in SYL.

Our estimates of LBP burden in Canada under the base-case scenario can also be compared with publicly available estimates of LBP burden from the GBD 2019. In spite of differences in data sources, methods of data processing and analysis, and different denominators, SYL-estimated back pain prevalence and back-related YLDs in Canada were comparable to the GBD estimates. For example, the LBP-related YLD rate per 100,000 in 2019 was 1,304 in the GBD and 1,572 in SYL.

### Exposure levels following interventions

The effects of interventions on average BMI, proportion exposed to occupational risks, and proportion participating in exercise following the interventions are shown in Figures A2-1 to A2-4 (Supplementary Materials). In 2040, depending on the level of intervention, the mean BMI in Canada for persons aged 20 years and older ranged from 25.2 to 22.2, proportion of adult Canadians exposed to occupational risk ranged from 54.6% to 0%, and proportion exercising ranged from 3.5% to 17.7%.

### YLDs averted

The YLD trajectories obtained from the microsimulation model for weight reduction, ergonomic interventions, and exercise participation are shown in Figures A2-5 to A2-7 (Supplementary Materials). The relationship between YLDs and average reduction in BMI was non-linear and a model with YLDs as a function of log-transformed BMI fit the data very well (R^2^ = 0.91) as shown in Figure A2-8 (Supplementary Materials). A 0.1 unit decrease in log-transformed BMI (9.5% decrease in BMI) per year over 20 years in those with BMI ≥ 25 would result in a reduction of 27,993 (95% CI 23,373, 32,614) YLDs between 2021 and 2040 (Table 2). In units of BMI, the effect ranged from 124,220 (-124,052, 372,492) YLDs averted for 0.1 units of BMI to 1,219,331 (984,868, 1,453,794) YLDs for 5 units of BMI (Table 3). This means that between 1.4% (-1.4, 4.1) and 13.5% (10.9, 16.0) of all YLDs due to LBP would be averted. Because the relationship followed a logarithmic curve, the additional impact per unit of BMI gradually diminished for larger reductions in BMI. Equivalent reductions in YLD rates per 1000 person-years are shown in Table A2-5 (Supplementary Materials).

The effect of ergonomic interventions on YLDs was proportional to the percentage of workers whose risk had been eliminated (R^2^ = 0.99 for a linear model) as shown in Figure A2-9 (Supplementary Materials). A one unit change (1%) in the proportion at-risk would change the YLDs by 19,416 (16,275, 22,557) over 20 years (Table 2). For the selected values of the intervention shown in Table 3, the effect ranged from 472,775 (173,701, 771,850) YLDs averted for an effective ergonomic intervention in 20% of workers to a theoretical maximum of 2,026,035 (1,701,643, 2,350,427) YLDs if all ergonomic risks were eliminated. Expressed in %, these reductions would be equivalent to between 5.2% (1.9, 8.5) and 22.4% (18.8, 25.9) of all YLDs due to LBP (Table 3). For specific occupational groups, the greatest impact, 821,773 (535,221, 1,108,324) YLDs averted, would be achieved by eliminating exposure in Group 7 (production and related workers, transport, equipment operators and labourers) because of its relatively large size; however, the greatest effect per person would be achieved in Group 6 (agriculture, animal husbandry, and forestry workers, fishermen, and hunters), in whom the relative risk was highest (Table 3).

The effect of exercise was proportional to the percentage of patients in the target group receiving the intervention (R^2^ = 0.99 for a linear model). This relationship is shown in Figure A2-10 (Supplementary Materials). On average, a one percent change in the proportion exercising would produce a difference of 26,058 (22,455, 29,661) YLDs (Table 2). The effects ranged from 597,534 (254,446, 940,622) YLDs averted if 20% of persons with back problems, excluding those aged 80 years and older and those with most severe pain, received the exercise intervention, to 2,682,177 (2,310,046, 3,054,308) if 100% persons in the target group received the intervention. These reductions translate to between 6.6% (2.8, 10.4) and 29.6% (25.5, 33.7) reduction in total LBP-related YLDs (Table 3). Using an alternative approach to calculating YLDs averted due to exercise (reduction in average pain on VAS rather than RR) resulted in almost identical effects (Table 3, Figure A2-10). Table A2-5 (Supplementary Materials) shows the effects expressed as reductions in YLDs per 1000 person-years.

### Equivalence of interventions

**Fig. 1:**
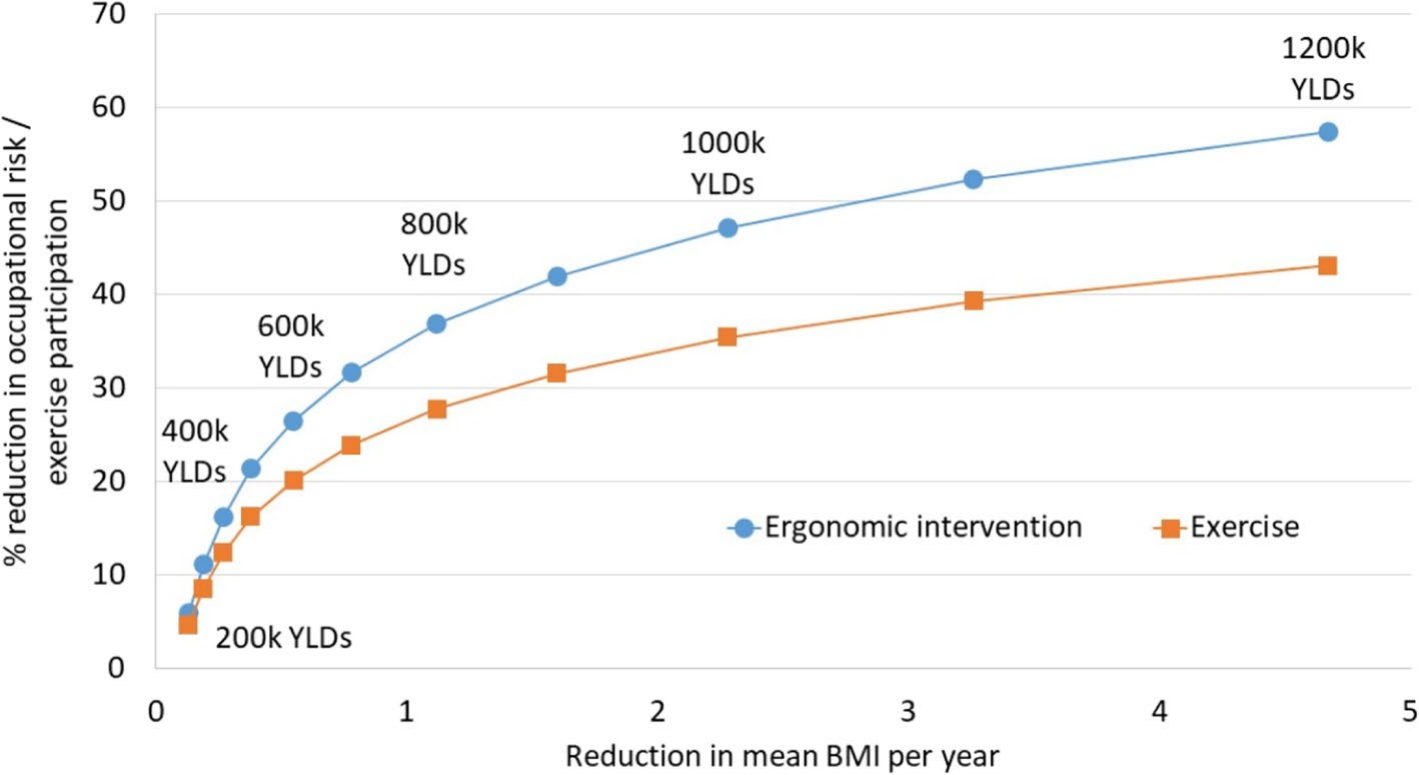
Equivalence between BMI, ergonomic and exercise interventions in terms of their impact on YLDs. Each pair of points represents a specific number of YLDs averted (in thousands). Values on the y-axis show the percent reduction in occupational risk in workers and the percent of eligible subjects participating in exercise that are required to achieve the same reduction in YLDs as the corresponding reduction in BMI shown on the x-axis. BMI: body mass index; YLDs: years lived with disability

Figure 1 shows the reduction in the proportion exposed to occupational risk and the proportion participating in an exercise program that would be YLD-equivalent to a given reduction in mean BMI per year. To reduce YLDs in Canada by 500,000 over 20 years would require a reduction in BMI of about 0.4 (0.2, 0.8) units per year among people who are overweight and those with obesity, an effective ergonomic intervention in 21.4% (6.2, 36.6) of workers, or exercise therapy in 16.3% (3.0, 29.5) of eligible patients with back problems. Interventions that could avert 1,000,000 YLDs would need to reduce BMI by 2.3 (1.1, 4.9) units per year, eliminate occupational risk of LBP in 47.2% (32.6, 61.7) of workers, or provide an exercise program to 35.4% (22.9, 48.0) of patients in the respective target groups.

## Discussion

In this study we projected and compared the potential impact of weight reduction, ergonomic interventions, and exercise on LBP-related YLDs in a population of a high-income country. According to our model, each of the interventions studied could result in a substantial reduction in disability. For example, a one unit reduction in BMI per year among people who are overweight and those who have obesity would avert about 770,000 YLDs over 20 years and would be approximately equivalent in terms of disability reduction to an effective ergonomic intervention in 35% of workers and an exercise intervention in 27% of eligible patients with back problems over the same period (Fig. 1).

To our knowledge this is the first population-based microsimulation study to compare currently available preventive strategies in low-back pain in terms of YLDs averted and to provide measures of equivalence between these interventions. We applied a microsimulation model of back pain developed with a new, user-friendly simulation platform (SYL). Our open population model was well calibrated and able to accurately represent the population of Canada over time, as evidenced by relatively small differences in the distribution of demographic and clinical variables between the CCHS and SYL populations (Table 1). In particular, the distribution of LBP in our simulation, derived from the 2001 cycle of the CCHS, remained very close to that observed in the 2016 cycle. Our model of LBP allowed us to follow individual trajectories in patients with back pain in a way consistent with empirical data and our contemporary understanding of the natural course of this condition. The level and trend in total LBP-related YLDs in the model between 2001 and 2019 was similar to the trend estimated for Canada by the GBD for the same period, despite differences in data sources and methods of estimation [3]. Further evidence of validity of our modeling strategy comes from the observation that our estimates of YLDs averted due to exercise were almost identical when using two different types of parameters, i.e., a relative risk of pain and absolute change in pain level, coming from two different meta-analyses.

The majority of previous simulation modeling studies of LBP were cost-effectiveness studies. Hall et al. reviewed 21 decision-analytical studies, including several surgical treatments (e.g., fusion, lumbar decompression, discectomy, artificial intervertebral disc replacement) and some pharmacological (pregabalin, duloxetine, NSAIDs) and non-pharmacological (acupuncture, heat) therapies for LBP and sciatica [22]. Most of the studies used a Markov or a decision tree model. Herman et al. applied a Markov model to study cost-effectiveness of 17 non-pharmacological therapies for LBP [23]. Others have studied cost-effectiveness of physiotherapy [24], cognitive behavioral therapy [25], or a stratified approach to treatment [26]. Markov models have also been used to estimate the costs of LBP in Sweden [27] and LBP-related work disability in the Netherlands [28].

Markov state transition and decision tree models can be very useful, but in situations when future events may be determined by prior states or events, or the number of health states required is large, microsimulation may be a preferred approach [22]. Microsimulation offers greater flexibility in specifying the statistical models describing event rates and changes in variables over time, ability to model many events simultaneously while considering competing risks, and modeling the distributions of variables in the population [29, 30]. A disadvantage is greater complexity, which tends to result in a longer computation time and may lead to less transparency and difficulties in fully accounting for all sources of uncertainty if the number of parameters is very large. One example of microsimulation modeling is a study by Schofield et al., who projected the national costs and productive years of life lost due to LBP in Australia from 2015 to 2030 [31].

Our aim was to assess the impact of preventive interventions on LBP burden in a realistic, open population over a long time horizon, and our model did not provide cost estimates. Therefore, it is generally not feasible to compare our data directly with the published cost-effectiveness studies. However, Herman et al. provided estimates of quality-adjusted live years (QALYs) gained for various non-pharmacological therapies, including exercise [23]. It is worth noting that despite differences in study objectives, populations modeled, data sources, and modeling methods, their results for the effect of exercise (13 to 33 QALYs gained per 1000 persons per year for different exercise programs) were similar to ours (20 to 93 YLDs averted per 1000 person-years, depending on percent participating). Herman et al. found yoga to be more effective (48 QALYs gained per 1000), but evidence for the effectiveness of non-pharmacological therapies other than exercise (with or without education) is weak [7, 11, 12].

Our approach to evaluating population impact of interventions was to consider a wide range of intervention levels (intensity or coverage) for each intervention type rather than attempting to determine a priori what level is most feasible or realistic. Whether a particular intervention is feasible or not may depend on many factors, some of which are difficult to identify or quantify. By deriving a relationship between intervention level and its impact on YLDs we believe the results are more useful and generalizable. To facilitate policy decisions, we provide a comparison of effectiveness in terms of YLD equivalence for a wide range of levels of the interventions studied.

Consider weight reduction, for example. Despite the enormous impact of the obesity epidemic, efforts to reduce obesity in Canada have not been successful. This, however, does not mean such reductions are not possible in the future, as average BMI has declined in some countries in the last decade [3]. Examples of effective obesity interventions include physician counselling, food labeling, fiscal measures, worksite interventions, mass media campaigns, food advertising regulation, and school-based interventions [32]. However, in the present study we were not interested in assessing and comparing the effects of these specific interventions. Rather, our focus was on the relationship between average BMI reduction (whatever the means for achieving it is) and population YLDs. In interpreting and using our results for policy decisions it is up to clinical experts, public health providers, and policymakers, in consultation with patients and representatives of the general public, to decide what level of weight reduction is achievable with the policies and approaches currently available. Furthermore, since new models in SYL can be developed rapidly, our modeling platform can be used interactively to assess the population health impact of any intervention of known efficacy.

Our study has several limitations. First, in selecting and implementing interventions to reduce LBP and the associated disability, policymakers may need to consider the costs of interventions. Although we did not estimate costs in the current study, our data can be useful in future cost-of-illness and cost-effectiveness studies. To this end, policy experts can generate cost estimates according to intervention intensity or coverage and apply our estimates of effect. Second, we modeled LBP using self-reported data from the CCHS. While LBP is a subjective phenomenon, self-reported weight tends to be underestimated [33]. Third, we simulated the population of Canada aged 20 years and older using a sample of about 100,000 individuals, matching the CCHS sample size. Although using a larger sample would reduce random error in the data, an advantage of our approach, in addition to computational efficiency, is that it allowed us to estimate the amount of error one would expect in a study of this size, and thus provide a measure of uncertainty in the estimates. Fourth, a limitation of every model is that the values of most parameters can be challenged and are estimated with error. The key parameters in our model are the effects of BMI, occupation, and exercise on the risk of LBP. The effect of BMI was obtained from an analysis of a very large survey, and was similar to the relative risk derived by others from a meta-analysis of published studies [19]. The effect of occupation was obtained directly from the GBD, and the effect of exercise was obtained from a recently published meta-analysis of randomized trials. For all these parameters, we provide 95% confidence intervals (Table A2-2, Supplementary Materials). Fifth, our model assumes that the effects of interventions on LBP risk are the same for both sexes, as separate estimates for males and females were not available. Sixth, we assumed that all persons diagnosed with chronic back problems would experience an episode of back pain at the time of diagnosis. If for some people this is not true, this assumption may cause inaccuracy in the results. Finally, our estimates are based on the assumption that changes in weight and ergonomic or exercise interventions will result in changes in LBP rates. At this time, there is evidence from randomized trials for the effectiveness of exercise [11, 12]. Evidence for the association of high BMI and occupational factors with LBP comes primarily from observational studies and a causal effect of modifying these factors has yet to be demonstrated experimentally [1–3, 7–10].

Generalizability of our results to other countries is an important question. It seems reasonable to assume that the relative risk estimates are generally applicable to most countries. We also believe that our base-case model of LBP, based on a very large, nationally representative Canadian survey, is probably generalizable to countries with a similar level of socio-demographic and economic development. Therefore, although LBP prevalence varies substantially by country, and the absolute YLDs also vary, relative measures of burden reduction are likely generalizable to high-income countries and potentially other countries as well. However, caution is needed when applying our data to low-income countries, where LBP is not as common and different burden reduction strategies may be needed [7].

## Supplementary Information

**Additional file 1:** **Appendix 1.** Technical description of SimYouLate. **Appendix 2.** Additional tables and figures.

## Data Availability

The datasets generated and/or analyzed during the current study are available from the corresponding author on reasonable request.

## Abbreviations

BMI: Body mass index
CCHS: Canadian Community Health Survey
GBD: Global Burden of Disease
LBP: Low back pain
LCL: Lower confidence limit
NOC-S: National Occupation Classification Statistics
NPHS: National Population Health Survey
NSAIDs: Non-steroidal anti-inflammatory drugs
OR: Odds ratio
QALYs: Quality-adjusted life years
RR: Relative risk
SYL: SimYouLate
UCL: Upper confidence limit
VAS: Visual analog scale
YLDs: Years lived with disability
