# Supplementary material for: Reducing the burden of low back pain: results from a new microsimulation model
Source: BMC Musculoskelet Disord. 2022 Aug 23;23:804. doi: 10.1186/s12891-022-05747-2 (PMC9396830; doi:10.1186/s12891-022-05747-2)
Supplement: Supplementary file 1 — Additional file 1: Appendix 1. Technical description of SimYouLate. Appendix 2. Additional tables and figures. [file 12891_2022_5747_MOESM1_ESM.doc]

# Appendix 1: Technical description of SimYouLate

**1.1 Overview**

SimYouLate (SYL) is a flexible, continuous-time microsimulation software. No data or models are hard-coded into SimYouLate. Rather, the researcher uploads an initial population data file, then uploads or specifies all the models in a given simulation. This removes the need for programmers (e.g., C++) when making changes to a simulation population or model. Simulation studies are typically quite iterative, where models are added, removed or changed based on intermediate runs. Not requiring a skilled programmer to modify complex code during this process presents a significant advantage over other simulation platforms.

The starting population with initial variables is loaded into SimYouLate as an Excel file. Options that control the simulation include start and end time “tick” (these are typically calendar years), random seed to allow replication of previous results (or else a clock-based random seed for new stochastic results each run), as well as an option for keeping or discarding temporary variables from the output (see below). It is important to note that if a researcher does not have a need for incremental time in his or her simulation (such as calendar year), the entire simulation can be run without any discrete ticks (or more precisely, between two ticks—entirely in continuous time).

Once the population data are loaded, models may be entered directly into the simulation by the analyst, using an open source, extremely flexible, generalized math parsing language. Available functions include: sine, cosine, tangent, arcsine, arccosine, arctangent, hyperbolic sine, hyperbolic cosine, hyperbolic tangent, hyperbolic arcsine, hyperbolic arccosine, hyperbolic arctangent, logarithm base 2, logarithm base 10, logarithm base e (2.71828...), e raised to the power of x, square root, sign function (-1 if x<0; 1 if x>0), round to nearest integer, absolute value, min of all arguments, max of all arguments, sum of all arguments, mean of all arguments, logical and, logical or, less or equal, greater or equal, not equal, equal, greater than, less than, addition, subtraction, multiplication, division, raise x to the power of y, and if-then-else operator, as well as any arbitrary combination/nesting of the above functions.

**1.2 Statistical models**

Statistical models can be uploaded in tables with formats common to the regression output from many statistical analysis packages (e.g., SAS). Supported models include (additional models can be added, as needed):

- Linear model: *
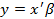
*
- Binary or ordinal logistic model:
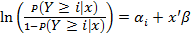

- Exponential event time model: probability density function
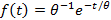
, where
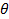
 is expected event time (inverse of hazard rate); regression models specified as
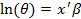

- Weibull event time model: probability density function , whereis the inverse of the scale parameter, and is the shape parameter; regression models can be specified separately for scale and shape parameters
- Cox proportional hazards event time model: , where
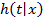
 is the hazard rate as a function of time, and is an unspecified baseline hazard function
- Stratified tables of hazard rates (e.g., mortality tables stratified by age and calendar year), uploadable directly into the simulation; treated as stratified exponential models.
- Error terms can be incorporated into the above models by way of random normal, uniform or other random variates

Models allow a simulation to perform deterministic calculations (e.g., age), randomly classify subjects on ordinal scales (e.g., Kellgren-Lawrence radiographic OA grade), generate competing events (e.g., joint replacement surgery vs. onset of diabetes vs. death). Interventions fall under the same umbrella; their effects can be input as risk modification variables (e.g., as a hazard ratio), while the intervention itself can be assigned to a subject according to calendar time (tick), and/or any number of other conditioning variables or functions (e.g., random assignment of intervention among at-risk individuals, weighted according to age, sex, BMI, or number of previous interventions).

Upon completion of the simulation, instead of only listing descriptive statistics as output, which may be too limited depending on the research question, SimYouLate output is comprised of an entire cross-sectional snapshot of the population at every time increment, all initial and derived variables, as well as the precise event times for all events that occurred. (It is important to note that events can occur at any time, and not generally on an actual discrete tick.)

**1.3 SimYouLate algorithm**

The SimYouLate simulation follows the following algorithm:

1. Start at the user-specified initial time tick. Cycle through models and hazard rates, calculating any linear functions (e.g., age = time - birth time), and defining any event time variables as 0 if they are derived variables, or else leaving unchanged if they preexisted on the population dataset. New events do not occur on the first cycle of output because each output cycle is defined as of the start of the given time cycle, so as of the first cycle output, 0 time has elapsed. Save the baseline time cycle in the output database. Increment time.
2. Cycle through the linear models, calculating any linear functions (e.g., age = time - birth time) that are set to occur at the START of a cycle.
3. Cycle through the event time models in the order they are defined, and draw random event times from the appropriate distributions (e.g., Weibull or exponential). Note these times do not yet assign events. Cycle through the hazard rate tables and do the same thing, drawing event times from an exponential distribution with the applicable hazard rate from the table. Compare event times obtained from models and tables. If the shortest time generated + an initial offset of 0 is T<1 time unit, define that event as having occurred at the current time tick + T (e.g., 2005.125 if time=2005 and T=.125), and write that event to the database. Add the event time to the offset, and repeat this step, redrawing new event times except for those events that already occurred. This is done because events that have just occurred may change the rate of remaining competing events (for example a diagnosis of osteoarthritis will increase the rate of joint replacement surgery). If the shortest time generated + the current offset is <1 time unit, define that event as having occurred at the given time + offset, and write it to the database. Add the time to the offset, and repeat, until the shortest generated time + current offset exceeds 1. At any time, if the special event DEATH occurs, stop generating events for the given subject. During every pass through the models described in this step, cycle through the linear models, recalculating any linear functions that are set to occur in REALTIME (for example, HALE, or health-adjusted life expectancy, can be more precisely calculated when intra-year changes in a measure of health are properly accounted for).
4. Cycle through the linear models again, calculating any linear functions that are set to occur at the END of the cycle.
5. Save a population snapshot at the completed time cycle in the output database. Increment time.
6. Repeat the above steps until all time ticks between initial time tick and ending time tick have been processed; in the case of an entirely continuous simulation, the above steps will not need to be repeated.

**1.4 SimYouLate examples of input**

1. **Hazard rates**

Tables of hazard rates (e.g., mortality rates available from Statistics Canada) are available in a variety of formats, and may be stratified by one to several variables (e.g., age, sex, province/state, calendar year). The table should first be transformed into a format with one hazard rate per row, and the stratification variables listed in separate columns. This table can be uploaded into the simulation. For example (the numbers in tables are given for illustration only):

| sex | obese | LBP | Rate |
| --- | --- | --- | --- |
| 1 | 0 | 0 | 0.00123 |
| 2 | 0 | 0 | 0.00210 |
| 1 | 1 | 0 | 0.01847 |
| 2 | 1 | 0 | 0.02537 |
| 1 | 0 | 1 | 0.13456 |
| 2 | 0 | 1 | 0.23625 |
| 1 | 1 | 1 | 0.31763 |
| 2 | 1 | 1 | 0.45252 |

(In practice, these tables may occupy several thousand rows or more.)

1. **Exponential event time model**

The Excel worksheet containing exponential regression coefficients should have 3 columns, labeled variables, levels and estimate. The variables column lists the independent variable(s). One row contains “Intercept”. Cells may list more than one variable, separated by * symbols, in the case of interactions. The levels column lists the level(s) associated with the variable(s) listed to the left. In the case of numeric variables, the level should be specified as “NUM”. In the case of an interaction term, the levels associated with the variables in the interaction should be listed respectively, and also separated by * symbols. The estimate column contains the regression coefficients. X*Beta in this model estimates ln(expected event time) in an exponential distribution, therefore exponentiated negative regression coefficients are hazard ratios.

For example:

| variables | levels | estimate |
| --- | --- | --- |
| Intercept | NUM | 3.14942523 |
| age | NUM | 0.048544951 |
| sex | 1 | -0.494282935 |
| sex | 2 | 0 |
| age*sex | NUM*1 | 0.014503588 |
| age*sex | NUM*2 | 0 |

1. **Weibull event time model**

Weibull event time models can be specified in a manner analogous to exponential regression, except that two linear predictors are specified separately, the first for the Weibull shape parameter and the second for the Weibull scale parameter.

1. **Proportional hazards event time model**

The Excel worksheet containing Cox proportional hazards model (PHM) regression coefficients should have 3 columns, labeled variables, levels and estimate. Format is similar to that described above for the exponential model, except in the PHM there is no intercept term. X*Beta in this model estimates ln(hazard ratio), therefore exponentiated regression coefficients are hazard ratios. For example:

| variables | levels | estimate |
| --- | --- | --- |
| LBP | 1 | -0.096564237 |
| sex | 2 | -0.011974358 |
| IncomeQuart | 2 | -0.000921722 |
| Income Quart | 3 | -0.051805909 |
| Income Quart | 4 | -0.046962355 |
| age | NUM | 0.020395688 |
| BMI | NUM | 0.000877222 |
| BMI*LBP*Income Quart | NUM*1*2 | 0.003340271 |
| BMI*LBP*Income Quart | NUM*1*3 | 0.003940673 |
| BMI*LBP*Income Quart | NUM*1*4 | 0.00524988 |

Note: The specified outcome variable must also be listed in another model, since the PHM does not specify a baseline hazard. It can be listed in a hazard rates sheet, an exponential model, or a Weibull model.

1. **Hazard ratios for event times**

The Excel worksheet containing hazard ratios should have 3 columns, labeled variables, levels and estimate. Format is similar to that described above for the proportional hazards model. There is no usual linear predictor X*Beta in this model; rather, hazard ratios from individual rows are evaluated and multiplied together.

For example:

| variables | levels | estimate |
| --- | --- | --- |
| LBP | 1 | 0.907951571 |
| sex | 2 | 0.98809705 |
| Income Quart | 2 | 0.999078703 |
| Income Quart | 3 | 0.949513141 |
| Income Quart | 4 | 0.954123315 |
| age | NUM | 1.020605101 |
| BMI | NUM | 1.000877607 |
| BMI*LBP*Income Quart | NUM*1*2 | 1.003345856 |
| BMI*LBP*Income Quart | NUM*1*3 | 1.003948448 |
| BMI*LBP*Income Quart | NUM*1*4 | 1.005263685 |

Note: The specified outcome variable must also be listed in another model, since the hazard ratios table does not specify a baseline hazard. It can be listed in a hazard rates sheet, an exponential model, or a Weibull model.

1. **Linear regression model**

The Excel worksheet containing linear regression coefficients should have 3 columns, labeled variables, levels and estimate. Format is similar to that described above for the exponential model. X*Beta in this model estimates the expected value of the dependent variable. For example:

| variables | levels | estimate |
| --- | --- | --- |
| Intercept | NUM | -24.89938574 |
| LBP | 1 | -0.359974724 |
| LBP | 0 | 0 |
| sex | 2 | 3.481972885 |
| sex | 1 | 0 |
| Income Quart | 2 | -0.238081263 |
| Income Quart | 3 | -0.115855331 |
| Income Quart | 4 | -0.026192454 |
| Income Quart | 1 | 0 |
| age | NUM | 0.280107656 |
| BMI | NUM | 0.50389217 |
| BMI*LBP*Income Quart | NUM*1*2 | 0.405425644 |
| BMI*LBP*Income Quart | NUM*1*3 | 0.520451748 |
| BMI*LBP*Income Quart | NUM*1*4 | 0.509710302 |
| BMI*LBP*Income Quart | NUM*1*1 | 0.388419615 |
| BMI*LBP*Income Quart | NUM*0*2 | 0.010600861 |
| BMI*LBP*Income Quart | NUM*0*3 | -0.01256875 |
| BMI*LBP*Income Quart | NUM*0*4 | -0.014789356 |
| BMI*LBP*Income Quart | NUM*0*1 | 0 |

1. **Logistic regression model**

The Excel worksheet containing ordinal or binary logistic regression coefficients should have 3 columns, labeled variables, levels and estimate. Format is similar to that described above for the exponential model, except in ordinal logistic regression there are multiple intercept terms, one for each level of the dependent variable minus one. X*Beta in this model represents the log odds of the cumulative outcome Y>=k, where k is the selected intercept number in calculating the linear predictor. For example (where Y is ordinal with values 0, 1, 2 and 3):

| variables | levels | estimate |
| --- | --- | --- |
| Intercept | 3 | -10.23382269 |
| Intercept | 2 | -9.495805324 |
| Intercept | 1 | -9.059432735 |
| LBP | 1 | 1.715546977 |
| sex | 2 | 0.434038266 |
| Income Quart | 2 | 0.380095145 |
| Income Quart | 3 | -0.044315633 |
| Income Quart | 4 | 0.123618171 |
| age | NUM | 0.050054929 |
| BMI | NUM | 0.106632141 |
| BMI*LBP*Income Quart | NUM*1*2 | -0.02811856 |
| BMI*LBP*Income Quart | NUM*1*3 | -0.003604519 |
| BMI*LBP*Income Quart | NUM*1*4 | -0.013758215 |

1. **Conditionals**

Conditionals can be specified for any model or formula to control who has the model applied to them and when. For example (in simplified syntax):

- If a specific intervention is planned for calendar years 2021 through 2025, the conditional might be specified as: time>=2021 && time <=2025
- If additionally, it is meant to target only obese women age 40+, the conditional might be specified as: time>=2021 && time <=2025 && sex==2 && BMI>=30 && age>=40
- If further, the campaign is expected to reach only a random 25% of the target demographic, the conditional might be specified as: time>=2021 && time <=2025 && sex==2 && BMI>=30 && age>=40 && RandUniform<.25
- Conditionals can also be used to ensure that interventions are limited to a pre-set maximum number (e.g., 5) per subject, for example: time>=2021 && time <=2025 && sex==2 && BMI>=30 && age>=40 && RandUniform<.25 && NumInterventions<=5
- Suppose that an additional target group is overweight men 50+, and the intervention is expected to reach 30% of that target demographic. Then the conditional might be: time>=2021 && time <=2025 && ((sex==2 && BMI>=30 && age>=40 && RandUniform<.25) OR (sex==1 && BMI>=25 && age>=50 && RandUniform<.3)) && NumInterventions<=5

**Appendix 2: Additional tables and figures**

Table A2-1. Description of the interventions.

| **Intervention** | **Description** | **Target population** |
| --- | --- | --- |
| Weight reduction | BMI is reduced by 0.1-1.0 units per year (in 0.1 increments) and by 1.5 to 5 units (in 0.5 increments) | All persons with BMI ≥25 |
| Ergonomic intervention in all workers | Occupational risk is reduced to the background (reference) level in 20%, 40%, 60%, 80%, and 100% of the target population | Workers in all occupational categories |
| Ergonomic intervention in selected groups | Occupational exposure is reduced to the background (reference) level in everyone in the target population | Workers in a given occupational category |
| Exercise (RR)^1^ | Proportion participating in exercise is 20%, 40%, 60%, 80%, and 100% of the target population | People <80 years old with back problems and pain level <4 |
| Exercise (VAS)^2^ | Proportion participating in exercise is 20%, 40%, 60%, 80%, and 100% of the target population | People <80 years old with back problems and pain level <4 |

The occupational groups are: 1 - Professional, technical and related workers, 2 - Administrative and managerial workers, 3 - Clerical and related workers, 4 - Sales workers, 5 - Service workers, 6 - Agriculture, animal husbandry, and forestry workers, fishermen and hunters, and 7 - Production and related workers, transport, equipment operators and laborers. BMI: body mass index; RR: relative risk; VAS: visual analog scale.

^1^ Effect expressed as a relative risk of pain;

^2^ Effect expressed as a difference in pain level.

Table A2-2. Key model parameters.

| **Parameter** | **Value (95% CI)** | **Source** |
| --- | --- | --- |
| Effect of BMI on LBP (OR) | 1.14 (1.11, 1.17) | CCHS^1^ |
| Effect of occupation on LBP (RR) | | GBD^2^ |
| Group 1 | 1.17 (1.07, 1.28) | GBD^2^ |
| Group 2 | 1.21 (0.96, 1.50) | GBD^2^ |
| Group 3 | 1.00 (1.00, 1.00) | GBD^2^ |
| Group 4/5 | 1.34 ((1.20, 1.51) | GBD^2^ |
| Group 6 | 3.78 (2.61, 5.31) | GBD^2^ |
| Group 7 | 1.54 (1.41, 1.68) | GBD^2^ |
| Effect of exercise on LBP (RR) | 0.71 (0.60, 0.83) | Published meta-analysis^3^ |
| Effect of exercise on LBP (0-100 VAS) | 10.00 (1.31 to 19.09) | Published meta-analysis^4^ |

The occupational groups are: 1 - Professional, technical and related workers, 2 - Administrative and managerial workers, 3 - Clerical and related workers, 4 - Sales workers, 5 - Service workers, 6 - Agriculture, animal husbandry, and forestry workers, fishermen and hunters, and 7 - Production and related workers, transport, equipment operators and laborers. BMI: body mass index; LBP: low back pain; CI: confidence interval; GBD: Global Burden of Disease; RR: relative risk; OR: odds ratio; VAS: visual analog scale; CCHS: Canadian Community Health Survey.

^1^ An ordinal logistic regression (proportional odds) model using data from the CCHS (2001)

^2^ GBD 2017 Risk Factor Collaborators. Global, regional, and national comparative risk assessment of 84 behavioural, environmental and occupational, and metabolic risks or clusters of risks for 195 countries and territories, 1990-2017: a systematic analysis for the Global Burden of Disease Study 2017. Lancet. 2018;392(10159):1923-1994.

^3^ Shiri R et al. Exercise for the prevention of low back pain: systematic review and meta-analysis of controlled trials. Am J Epidemiol. 2018;187(5):1093-1101.

^4^ Chou R et al. Noninvasive treatments for low back pain. Agency for Healthcare Research and Quality (AHRQ), U.S. Department of Health and Human Services. AHRQ Publication No. 16-EHC004-EF, February 2016.

Table A2-3. Mapping of the GBD occupational categories to the CCHS occupational categories.

| **GBD categories** | **CCHS categories** |
| --- | --- |
| 1. Professional, technical and related workers | 2. Professional (including accountants)  3. Technologist or technician |
| 2. Administrative and managerial workers | 1. Management |
| 3. Clerical and related workers | 4. Administrative, financial or clerical |
| 4. Sales workers  5. Service workers | 5. Sales or service |
| 6. Agriculture, animal husbandry, and forestry workers, fishermen and hunters | 7. Farming, forestry, fishing, mining |
| 7. Production and related workers, transport, equipment operators and labourers | 6. Trades, transport or equipment operator  8. Processing, manufacturing, utilities |
| 8. Background (reference) | 9. Other |

GBD: Global Burden of Disease; CCHS: Canadian Community Health Survey.

Table A2-4. Mapping of the GBD low back pain disability weights onto HUI3 pain levels.

| **GBD description** | **Details** | **HUI3 description** | **Disability weight** |
| --- | --- | --- | --- |
| Mild | This person has mild back pain, which causes some difficulty dressing, standing, and lifting things. | Mild to moderate pain that prevented no activities | 0.020 (0.011, 0.035) |
| Moderate | This person has moderate back pain, which causes difficulty dressing, sitting, standing, walking, and lifting things | Moderate pain that prevented a few activities | 0.054 (0.035, 0.079) |
| Severe without  leg pain | This person has severe back pain, which causes difficulty dressing, sitting, standing, walking, and lifting things. The person sleeps poorly and feels worried. | Moderate to severe pain that prevented some activities (% with leg pain obtained from the GBD^1^) | 0.272 (0.182, 0.373) |
| Severe with leg pain | This person has severe back and leg pain, which causes difficulty dressing, sitting, standing, walking, and lifting things. The person sleeps poorly and feels worried. | Moderate to severe pain that prevented some activities (% with leg pain obtained from the GBD^1^) | 0.325 (0.219, 0.446) |
| Most severe without leg pain | This person has constant back pain, which causes difficulty dressing, sitting, standing, walking, and lifting things. The person sleeps poorly, is worried, and has lost some enjoyment in life. | Severe pain that prevented most activities. (% with leg pain obtained from the GBD^1^) | 0.372 (0.250, 0.506) |
| Most severe with leg pain | This person has constant back and leg pain, which causes difficulty dressing, sitting, standing, walking, and lifting things. The person sleeps poorly, is worried, and has lost some enjoyment in life. | Severe pain that prevented most activities (% with leg pain obtained from the GBD^1^) | 0.384 (0.256, 0.518) |

GBD: Global Burden of Disease; HUI3: Health Utilities Index Mark 3.

^1^ GBD 2017 Risk Factor Collaborators. Global, regional, and national comparative risk assessment of 84 behavioural, environmental and occupational, and metabolic risks or clusters of risks for 195 countries and territories, 1990-2017: a systematic analysis for the Global Burden of Disease Study 2017. Lancet. 2018;392(10159):1923-1994.

Table A2-5. YLDs averted per 1000 person-years among adults in Canada between 2021 and 2040 for three types of interventions.

| **Intervention** | **YLDs averted per 1000 person-years, 95% CI** | | |
| --- | --- | --- | --- |
|  | **Estimate** | **LCL** | **UCL** |
| Mean BMI reduction per year | | | |
| 0.1 | 4.3 | -4.2 | 12.8 |
| 0.3 | 15.0 | 7.1 | 22.9 |
| 0.5 | 20.0 | 12.3 | 27.8 |
| 1.0 | 26.8 | 19.2 | 34.5 |
| 2.0 | 33.6 | 25.9 | 41.3 |
| 3.0 | 37.6 | 29.7 | 45.4 |
| 4.0 | 40.4 | 32.4 | 48.3 |
| 5.0 | 42.6 | 34.5 | 50.6 |
| Reduction in occupational risk for all groups combined | | | |
| 20% | 16.3 | 6.9 | 25.7 |
| 40% | 29.8 | 20.8 | 38.8 |
| 60% | 43.3 | 34.4 | 52.3 |
| 80% | 56.8 | 47.4 | 66.2 |
| 100% | 70.4 | 60.2 | 80.6 |
| Elimination of occupational risk by occupational group | | | |
| Group 1 | 16.6 | 7.2 | 25.9 |
| Group 2 | 6.7 | -3.2 | 16.7 |
| Group 4&5 | 26.1 | 17.1 | 35.2 |
| Group 6 | 18.6 | 9.3 | 27.9 |
| Group 7 | 28.3 | 19.3 | 37.3 |
| Exercise participation (effect measured by RR) | | | |
| 20% | 20.4 | 10.8 | 30.1 |
| 40% | 38.6 | 29.4 | 47.8 |
| 60% | 56.7 | 47.5 | 65.9 |
| 80% | 74.9 | 65.2 | 84.5 |
| 100% | 93.0 | 82.6 | 103.5 |
| Exercise participation (effect measured by VAS) | | | |
| 20% | 20.7 | 14.5 | 26.9 |
| 40% | 40.0 | 34.0 | 45.9 |
| 60% | 59.2 | 53.3 | 65.1 |
| 80% | 78.4 | 72.2 | 84.6 |
| 100% | 97.6 | 90.9 | 104.4 |

The occupational groups are: 1 - Professional, technical and related workers, 2 - Administrative and managerial workers, 3 - Clerical and related workers, 4 - Sales workers, 5 - Service workers, 6 - Agriculture, animal husbandry, and forestry workers, fishermen and hunters, and 7 - Production and related workers, transport, equipment operators and laborers. Group 3 is not shown individually because RR = 1.0 in this group. YLDs: years lived with disability; BMI: body mass index; RR: relative risk; VAS: visual analog scale; LCL: lower 95% confidence limit; UCL: upper 95% confidence limit.


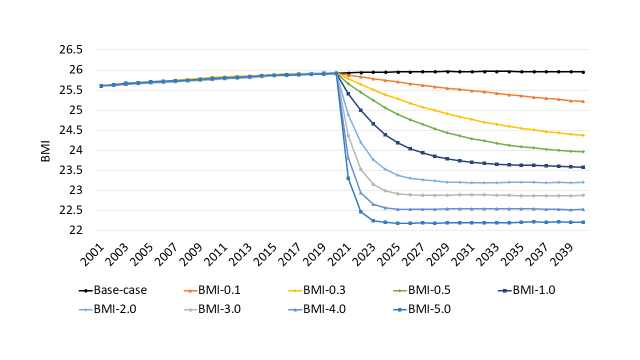


Figure A2-1. Mean BMI in the simulated population according to average reduction in BMI per year among persons who are overweight or have obesity. BMI: body mass index.


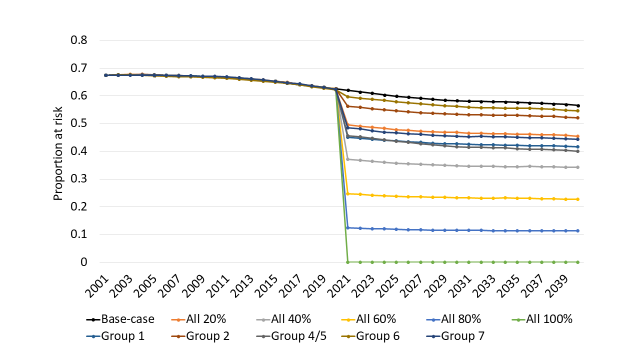


Figure A2-2. Proportion of adult Canadians exposed to occupational risk of LBP according to intervention level and target occupational group, before and after an ergonomic intervention. Group 3 is included in the overall proportion, but not individually since RR = 1.0 for this group. The occupational groups are: 1 - Professional, technical and related workers, 2 - Administrative and managerial workers, 3 - Clerical and related workers, 4 - Sales workers, 5 - Service workers, 6 - Agriculture, animal husbandry, and forestry workers, fishermen and hunters, and 7 - Production and related workers, transport, equipment operators and laborers. LBP: low back pain; RR: relative risk.


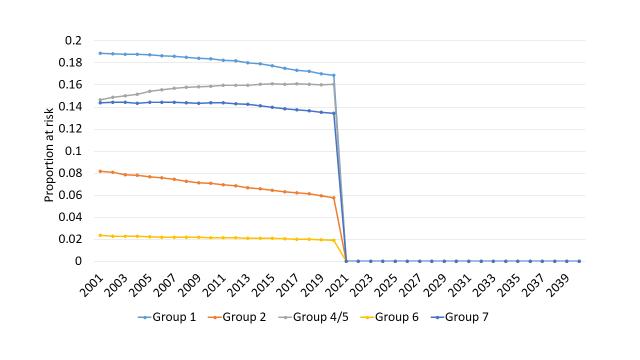


Figure A2-3. Proportion of adult Canadians exposed to occupational risk of LBP for each occupational group individually, before and after an ergonomic intervention. We assume that occupational risk is eliminated in all workers in a given category. Group 3 is not shown because RR = 1.0 in this group. The occupational groups are: 1 - Professional, technical and related workers, 2 - Administrative and managerial workers, 3 - Clerical and related workers, 4 - Sales workers, 5 - Service workers, 6 - Agriculture, animal husbandry, and forestry workers, fishermen and hunters, and 7 - Production and related workers, transport, equipment operators and laborers.


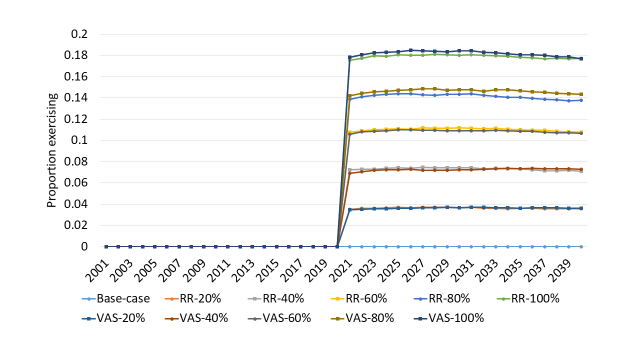


Figure A2-4. Proportion of adult Canadians with back problems, participating in an exercise program before and after the exercise intervention by intervention level, using relative risk (RR) and absolute reduction (VAS) scales for defining the effect of exercise on LBP. RR: relative risk; VAS: visual analog scale.


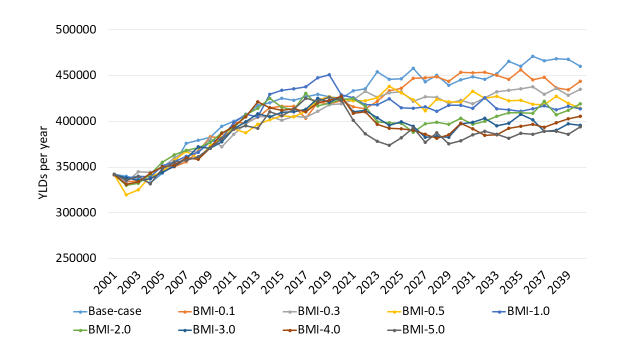


Figure A2-5. Trajectories of total LBP-related YLDs over time in persons 20 years of age and older in Canada from 2001 to 2040 according to the level of weight reduction intervention. Estimates from the SYL model. BMI: body mass index; LBP: low back pain; YLDs: years lived with disability.


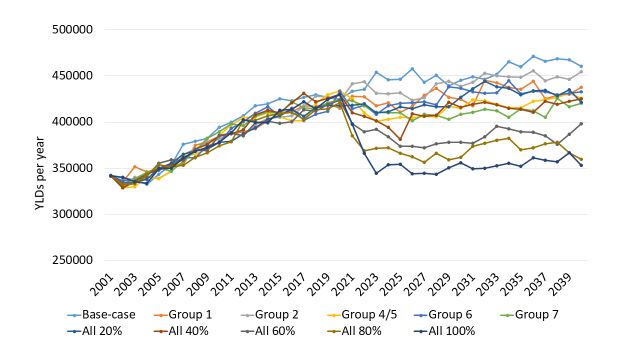


Figure A2-6. Trajectories of total LBP-related YLDs over time in persons 20 years of age and older in Canada from 2001 to 2040 according to percent reduction in occupational risk and target occupational group. Estimates from the SYL model. Group 3 is not shown because RR = 1.0 in this group. The occupational groups are: 1 - Professional, technical and related workers, 2 - Administrative and managerial workers, 3 - Clerical and related workers, 4 - Sales workers, 5 - Service workers, 6 - Agriculture, animal husbandry, and forestry workers, fishermen and hunters, and 7 - Production and related workers, transport, equipment operators and laborers. LBP: low back pain; YLDs: years lived with disability.


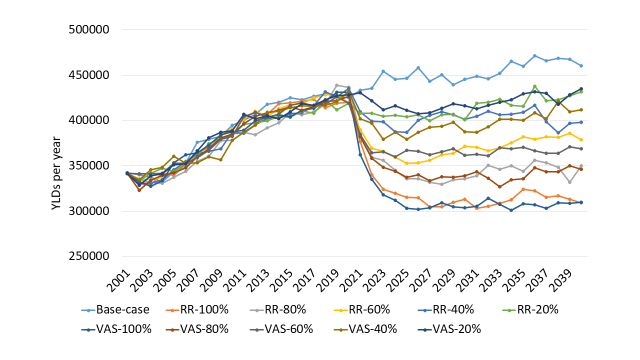


Figure A2-7. Trajectories of total LBP-related YLDs over time in persons 20 years of age and older in Canada from 2001 to 2040 according to exercise participation (%) and type of parameter (RR or VAS). Estimates from the SYL model. RR: relative risk; VAS: visual analog scale. LBP: low back pain; YLDs: years lived with disability.


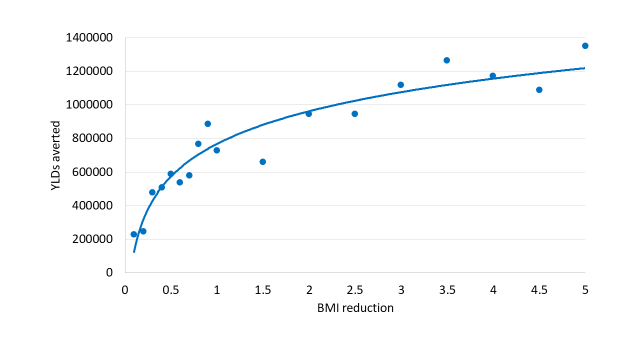


Figure A2-8. Relationship (log-linear model) between mean BMI reduction per year and YLDs averted among persons aged 20+ with BMI ≥25 in Canada between 2021 and 2040. YLDs: years lived with disability; BMI: body mass index.


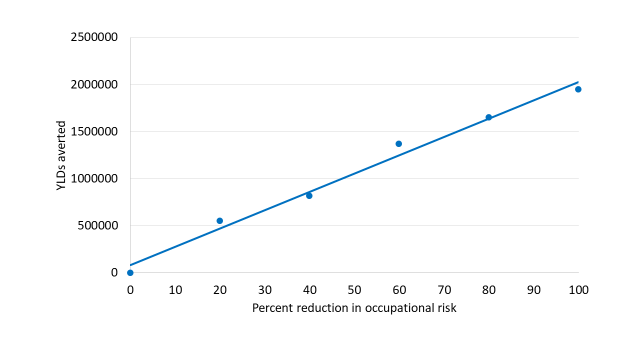


Figure A2-9. Relationship (linear model) between percent reduction in occupational risk and YLDs averted among persons aged 20+ in Canada between 2021 and 2040. YLDs: years lived with disability.


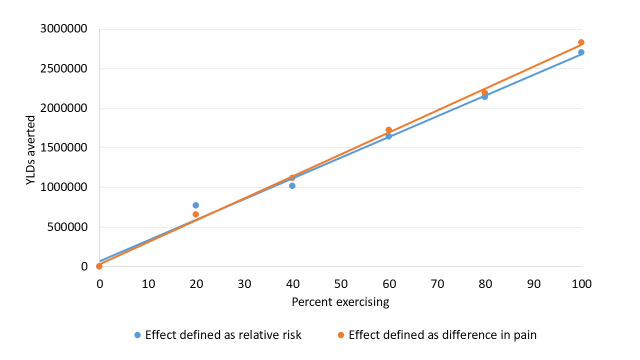


Figure A2-10. Relationship (linear model) between exercise participation and YLDs averted among persons aged 20+ in Canada between 2021 and 2040. The effect of exercise is expressed as a relative risk of pain and a difference in pain level. YLDs: years lived with disability.
